# Fostering development of nursing practices to support integrated care when implementing integrated care pathways: what levers to use?

**DOI:** 10.1186/s12913-017-2687-0

**Published:** 2017-11-29

**Authors:** Caroline Longpré, Carl-Ardy Dubois

**Affiliations:** 1 Faculty of Nursing, University of Montreal, Montreal, Quebec Canada; 2 Department of Nursing, University of Quebec, Outaouais, St-Jerome, Quebec, Canada

**Keywords:** Care integration, Organizational resources, Clinical-administrative processes, Professional practice, Nursing, Change strategies

## Abstract

**Background:**

Care integration has been the focus of recent health system reforms. Given their functions at all levels of the care continuum, nurses have a substantial and primordial role to play in such integration processes. The aim of this study was to identify levers and strategies that organizations can use to support the development of a nursing practice aligned with the requirements of care integration in a health and social services centre (HSSC) in Quebec.

**Methods:**

The research design was a cross-sectional descriptive qualitative study based on a single case study with nested levels of analysis. The case was a public, multi-disciplinary HSSC in a semi-urban region of Quebec. Semi-structured interviews with 37 persons (nurses, professionals, managers, administrators) allowed for data saturation and ensured theoretical representation by covering four care pathways constituting different care integration contexts. Analysis involved four steps: preparing a predetermined list of codes based on the reference framework developed by Minkman (2011); coding transcript content; developing general and summary matrices to group observations for each care pathway; and creating a general model showing the overall results for the four pathways.

**Results:**

The organization’s capacity for response with regard to developing an integrated system of services resulted in two types of complementary interventions. The first involved investing in key resources and renewing organizational structures; the second involved deploying a series of organizational and clinical-administrative processes. In resource terms, integration efforts resulted in setting up new strategic services, re-arranging physical infrastructures, and deploying new technological resources. Organizational and clinical-administrative processes to promote integration involved renewing governance, improving the flow of care pathways, fostering continuous quality improvement, developing new roles, promoting clinician collaboration, and strengthening care providers’ capacities. However, progress in these areas was offset by persistent constraints.

**Conclusions:**

The results highlight key levers organizations can use to foster the implementation and institutionalization of integrative nursing practices. They show that progress in this area requires a combination of strategies using multiple complementary levers. They also suggest that such progress calls for rethinking not only the deployment of certain organizational resources and structures, but also a series of organizational and clinical processes.

## Background

Care integration has been a core priority of recent health system reforms and has often been considered as a solution for reorganizing health and social services to ensure greater accessibility, to reinforce coordination, continuity, and quality, and to respond more effectively to population needs. In the province of Quebec, governments and health institutions have promoted various clinical and organizational methods, such as merging institutions, organizing activities into programs, strengthening links between primary and secondary care, developing care pathways tailored to the needs of specific patients, organizing services into hierarchical structures based on principles of population-based responsibility—all strategies that have been associated with stronger integration of care and services [1]. A study has suggested, however, that the transformations sought from these different strategies, in both the professional and organizational arenas, are often slow to materialize [2].

Given their functions in care provision at all levels of the care continuum, nurses have a substantial role to play in these transformation processes [3–5]. To contribute optimally to these processes, nurses’ roles, knowledge, competencies, and practices need to be renewed and adapted to new clinical and management requirements [5]. However, a recent analysis revealed gaps and significant delays in implementing nursing practices that could contribute to care integration. These delays were observed in various dimensions of practice: knowledge about the roles and responsibilities of different care providers; formalized collaboration processes and agreements; communication mechanisms and collaboration practices; support for interdisciplinary approaches; and support for team work [2].

At the organizational level, successive changes introduced by governments have often succeeded in transforming administrative structures without actually producing new forms of governance, fundamentally innovative management practices, or radically different ways of organizing services [6]. Consequently, the problems that prompted these changes remain unresolved: deficiencies in accessibility, continuity, and complementarity of care and services; excessive use of hospital-based services; and overcrowding of emergency rooms [7].

These difficulties in achieving the transformations of professional and organizational practices that are often announced in service integration projects reflect the numerous issues and challenges associated with such changes. For nurses, these changes involve developing new functions and roles, acquiring new competencies, and collaborating more closely with other professionals, while negotiating the boundaries of their activities with these professionals. Very few studies have documented the views of nurses directly involved in these processes and the factors that facilitate or impede the changes needed in nursing practice.

Depending on the care pathway, important variations can be observed in clinical practices, reflecting contrasting ways of implementing mechanisms for care integration [2], as well as an association between nurses’ well-being and the integration processes and their perceptions of these processes [8]. Given these observations, identifying the factors that facilitate or hinder nursing practice in an integration context seems particularly relevant.

### Goals and objectives

Given nurses’ perspectives as front-line actors, the aim of this study was to identify the levers and key strategies that organizations can use, in terms of management and clinical practice, to support the development of a nursing practice that is aligned with the requirements of care integration in a health and social services centre (HSSC) in Quebec. Specifically, the study had two objectives, which were to identify, in light of the main stakeholders’ experiences: 1) what factors support the practice of nurses in an integrated care setting; and, 2) what factors constitute barriers to nursing practice in this context.

### Conceptual and theoretical bases

Numerous studies have been devoted to modelling integrated care systems, resulting in various models. Among these are the Chronic Care Model [9], the Development Model for Integrated Care (DMIC), [10], the Kaiser Permanente model (https://healthy.kaiserpermanente.org/), and the theory of integration of care for frail seniors [11]. The implementation conditions for these models have generally received less attention. However, the relevance of implementation context for these models is justified based on organizational literature recognizing that contexts influence the way in which organizations structure themselves, operate, and produce results [12]. For Champagne & al. (2009) [13], analyzing the implementation of models serves to study how organizational and contextual factors influence the introduction of innovations in the configuration of services and the subsequent results of the services. Scott, Ruel, Mendel, and Caronna (2000) [14], for example, pointed to the impact of the material environment (e.g. availability of resources and technologies) and institutional environment (e.g. standards, values, and governance system) on the implementation of innovation. Other studies have examined the impact of cultural environments (e.g. beliefs, values, and norms); technical environments (e.g. actors’ abilities); strategic environments (e.g. strategic priorities); and structural environments (e.g. the resources available to the organization and the structures it has put in place to carry out its mission) [13].

**Fig. 1:**
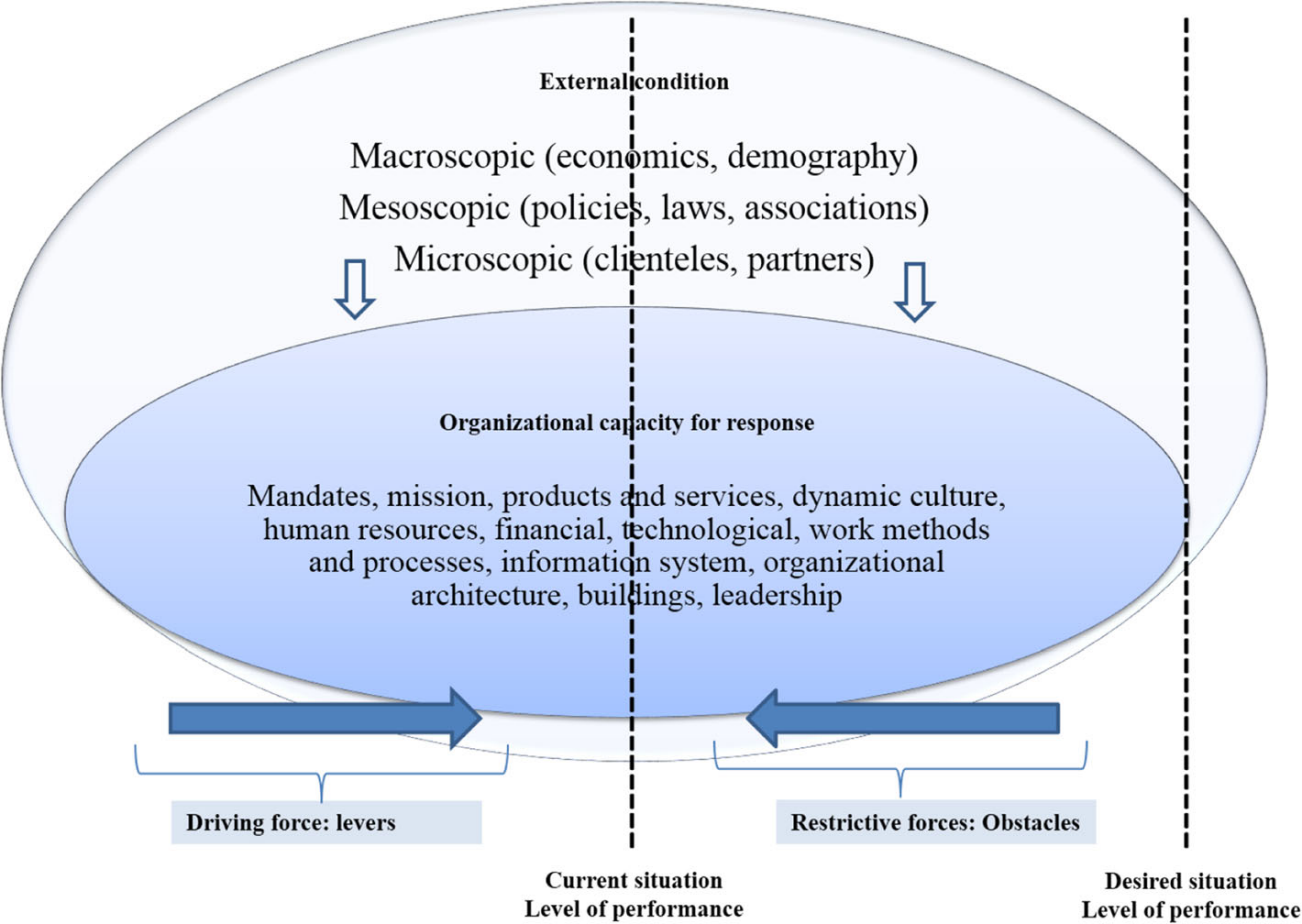
Reference framework. Adapted from Collerette & al. (2013), Lewin (1951), and Minkman (2011)

Collerette, Lauzier, and Schneider’s model of strategic organizational analysis [15] provides an integrative framework that synthesizes these different factors (Fig. 1). The model was developed based on a systemic perspective, according to which organizations must adapt to their environments to achieve their objectives [16]. The systemic perspective involves studying an object in all of its complexity. According to this perspective, an organization’s health depends largely on its ability to adapt to the characteristics, requirements, and challenges of its environment. It is a question of apprehending the object in its environment, its functions, its mechanisms of action, its structure, and its evolution [17].

Relying on this perspective enables the framework proposed by Collerette & al. [15] to take into account a wide array of factors that operate at various levels, interact with each other, and are likely to influence nursing practice in an integration context. According to the framework, these changes in practice are influenced not only by a set of external factors (demographic and epidemiological changes; technological developments; economic, political and social contexts, etc.), but also by a set of internal organizational conditions that represent an organization’s capacity to respond to external circumstances.

Collerette & al.’s framework [15] is further enhanced by Lewin’s (1951) ‘force-field theory’, [18] according to which a social situation (seen as dynamic) is characterized as the result of the combined effect of multiple forces in a more or less stable state of equilibrium. These can be driving forces or levers that exert a positive influence in the direction of the desired change, or restrictive or constraining factors that oppose the change [15, 19]. Factors operating at different levels can thus generate both driving and restrictive forces, working for or against the desired change. By using Collerette & al.’s framework [15], this study hopes to open what has until now been a black box and identify, drawing on nurses’ own experiences, the organizational factors that act as levers or constraints with respect to developing their practice in a care integration context.

## Methods

### Design

The research design was a cross-sectional descriptive qualitative study based on a single case study with nested levels of analysis [20].

### Study environment

The case studied was that of a public, multi-activity and multi-disciplinary health and social services centre (HSSC) located in a semi-urban region of Quebec, which provides services to more than 280,000 inhabitants (a population characterized by a higher percentage of aging individuals and a higher birth rate than the Quebec average). Using a program-based approach, the HSSC coordinates services provided by four local community health centres (CLSCs), four long-term care facilities (CHSLDs), an average-sized hospital, and more than 200 partners providing various services (community pharmacies, family medicine groups [FMGs], community organizations, group homes, etc.). This HSSC was selected as the locus for the study because it faced challenges similar to those confronting the great majority of HSSCs in Quebec: administrative authority over a large territory; a population with disparate socio-economic characteristics; rapid increase in population needs for health services; human resource shortages; and budget constraints.

With the support of key participants (directors of service programs), four care pathways were selected. A care pathway aims at a multidisciplinary, optimal, and consensual management of patients presenting the same pathology or the same care situation. Patients follow a clinical path that provides timely access to the services they need in a coordinated manner. Shared responsibility of institutions or professionals towards a patient requires integration of care. Pathways were selected based on the following criteria: feasibility of studying the pathway; management interest and availability; inclusion of pathways at different stages of development; and coverage of different care integration contexts. The care pathways selected were: chronic obstructive pulmonary disease (COPD), palliative oncology services (POS), autonomy support for the elderly (ASE), and mental health services (MHS). A previous study that evaluated so-called ‘integrative’ practices in this organization showed that the COPD and MHS pathways were at less advanced phases of integration (Phase 1 – initiative and design), while the ASE and POS pathways were at intermediate phases (Phase 2 – experimentation and execution, and Phase 3 – expansion and monitoring, respectively), and that none of the pathways had reached Phase 4 (consolidation and transformation), the most advanced phase of the integration process [2].

### Data sources

The strategy used was purposive sampling [20] from a maximal variety of sources: members of the nursing team (nursing assistant, nurse technician and clinician, nurse navigator, liaison nurse, care consultant), members of the interprofessional team (respiratory therapist, social worker, occupational therapist, physical therapist), managers, and senior administrators. Nurses and other professionals were selected with the assistance of managers and program directors based on the following inclusion criteria: French-speaking; holder of a practice license from a professional association; employed in one of the targeted care pathways for at least the previous six months (full- or part-time, days or nights, or on rotation); interested and available to participate in the study. Managers and senior administrators were selected based on the following criteria: French-speaking; employed full-time in the organization for at least the past six months; interested and available to participate in the study. All the people approached (*n* = 37) agreed to participate (100%); this sample size not only allowed for data saturation, but also ensured theoretical representation by covering four care pathways representing different care integration contexts, as well as the different perspectives of clinicians and managers. Respondents were evenly distributed across the four pathways: ASE (*n* = 7), MHS (*n* = 10), POS (*n* = 9), and COPD (*n* = 9). They included all nursing job titles (nursing assistant, nurse technician, nurse clinician, nurse navigator, nurse practitioner, nurse advisor, nurse manager) (*n* = 27), other professionals (*n* = 4), two representatives of executive management (*n* = 2), and four managerial or administrative representatives with an overall view of the care pathways—one for each pathway (*n* = 4). The objective of obtaining this diversity of perspectives was to increase the validity of the study results and improve our understanding of the phenomenon being studied [21].

The great majority of respondents were women (*n* = 32), had university degrees (*n* = 31), and worked days (*n* = 34). Participants were spread across different missions of the organization: hospital (*n* = 14), CLSCs (*n* = 15), CHSLDs (*n* = 4), and FMGs (*n* = 2). Sample composition was also balanced between management (*n* = 18) and clinical (*n* = 18) functions.

### Data collection process

This project was approved by the research ethics committee of the University of Montreal, the research ethics and administrative committees of the study sites, and the HSSC senior management team. An information letter was hand-delivered to the targeted participants and its contents were explained to them. All interviews were conducted by the lead investigator as part of a doctoral study. Interviews were booked directly with each respondent, for confidentiality purposes and to accommodate their preferred time and place. Interviews lasted approximately 60 min. Participants signed a consent form authorizing both audio-recording of the interviews and written note-taking [21]. A literature review was also conducted.

### Data collection instruments

Two data collection instruments were used: 1) an interview guide for nurses, managers, and professionals either working in the four care pathways or involved in their management, to collect their perceptions of the restrictive and facilitative forces influencing the adoption of nursing practices to support integrated care. The interview guide for nurses and professionals contained questions oriented toward clinical practice, while the interview guide for managers, directors, and administrators included an additional section covering administrative issues that could be used to describe care trajectories administratively. 2) a sociodemographic questionnaire (sex, employment position, training, work location and shift, seniority). Documents recommended by key participants provided additional relevant information, including, among other things, the organizational structure of the HSSC, organizational policy statements relating to nursing, and the HSSC’s management principles, strategic plans, and annual reports.

### Data analysis

All audio-recordings of the interviews were fully transcribed. Verbatim transcription of the interviews resulted in an average of 50 pages of text per interview, for a total of around 1850 pages (37 interviews of 50 pages). The principal investigator drafted a five-page summary for each interview, which was then submitted to the corresponding respondent for content validation [21]. Out of the 37 respondents, 18 confirmed the contents as accurate, four suggested minor adjustments due to changes in their situation, and the remainder did not follow up.

**Fig. 2:**
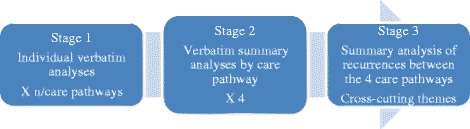
Analyse process

To ensure the validity of the coding process, the summaries and coding for five interviews were subjected to intra- and inter-rater validation. The intra-rater validation involved having the same researcher repeat the coding, after a one-week interval. A 90% similarity score was obtained between the two codings. The inter-rater coding process was carried out with the lead investigator, an expert in qualitative process and content. The validation process continued until 80% agreement among codings was obtained. This pre-coding exercise was used to validate the process, after which all the transcripts were coded. Following transcript validation, the data were analyzed in three main steps (Fig. 2). NVivo 10 software was used to systematically organize the coded data and to develop themes and subthemes, as well as conceptual matrices.

In the first step, the coding process was based on the dimensions of Minkman’s (2011) model of integrative practice [10] and the framework of Collerette & al.’s strategic organizational analysis model [15]. The verbatim content was coded in three steps: 1) according to the dimension of nursing practice that it represented; 2) according to the dimension of organizational capacity for response to which it corresponded; and 3) according to whether it expressed a driving force or a restrictive force. In essence, for each dimension of practice, analyzing the forces and obstacles using the Collerette & al.’s model helped identify recurrences and group content. For example, for content identified as part of the ‘multidisciplinary practice’ dimension (a theme) [10], elements related to human and physical resources (subthemes) [15] were identified and in turn gave rise to categories of driving forces and restrictive forces (third-level theme). Table 1 (below) provides an example illustrating the basic outline of the analysis.

**Table 1:** Basic outline of the analysis

Verbatim	Theme (Minkman)	Subtheme (Collerette)	Third-level: Force
“We use the AINÉS tool to help us better plan our report, and that helps us become familiar with our place in the interdisciplinary team”	Interdisciplinarity (support for collaboration)	Training	Driving force present in the organization: Clinical tool facilitating the report.

In the second step, summaries were developed to group the observations for each care pathway. Using the themes and subthemes as a starting point, analysis of the respondents’ verbatim statements regarding each care pathway helped identify the different elements that arose as driving or restrictive forces in a care pathway. Table 2 presents the driving and restrictive forces and the subthemes corresponding to the main theme ‘Support for collaboration’ for each care pathway.

**Table 2:**
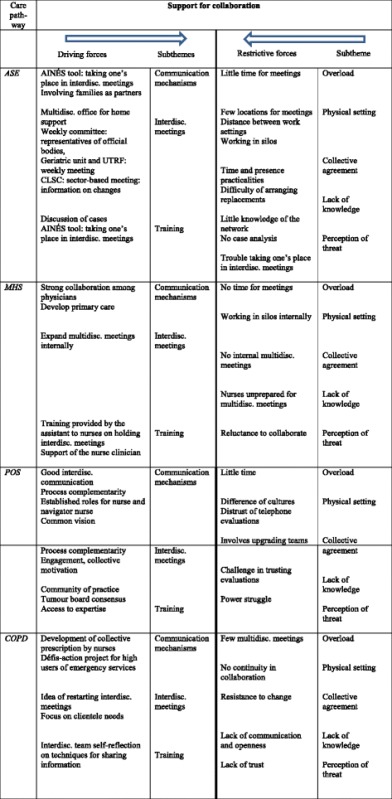
Synthesis of information on the theme ‘Support for collaboration’

**Fig. 3:**
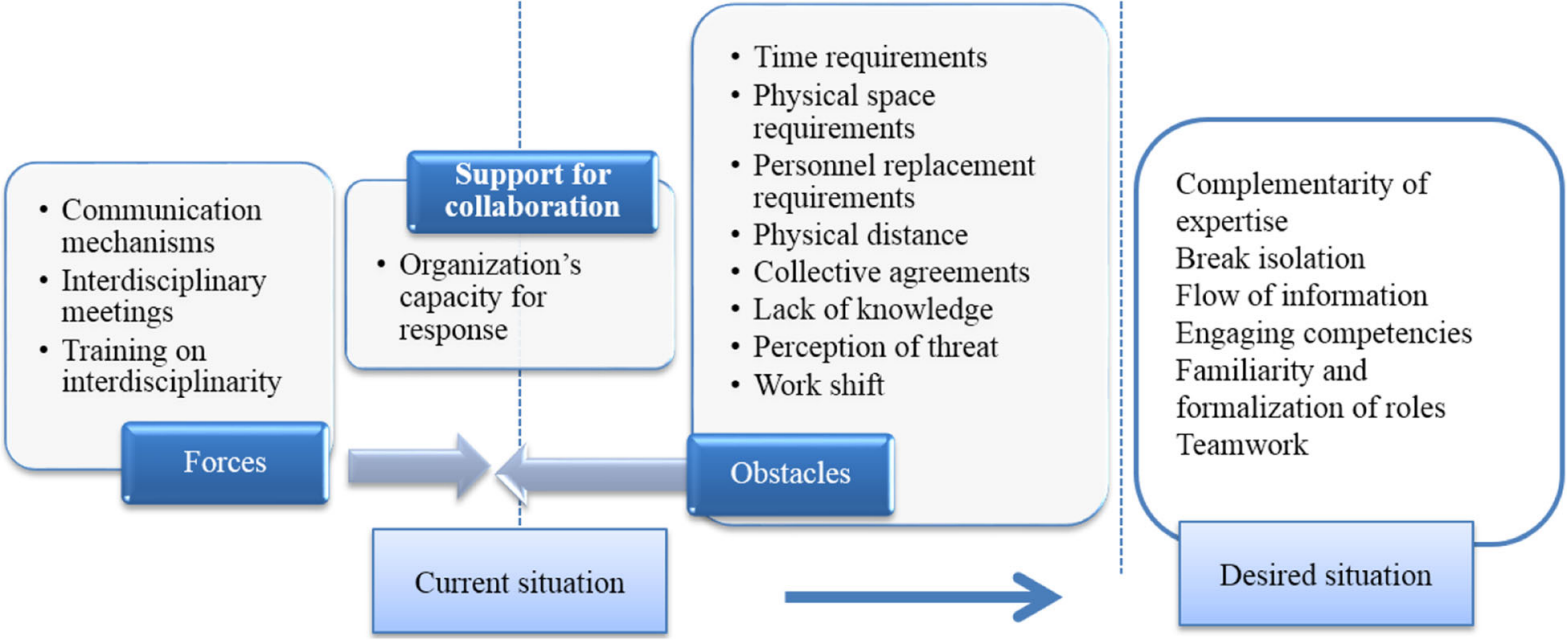
Matrix: supporting collaboration

In the third step, the analysis consisted in bringing together the cross-cutting results obtained from observation of the four pathways. For example, for the theme ‘Support for collaboration’, the subtheme ‘training’ was identified across all four care pathways. Figure 3 presents the resulting matrix, which consolidates data from the four care pathways with respect to the driving and restrictive forces for this theme.

## Results

**Fig. 4:**
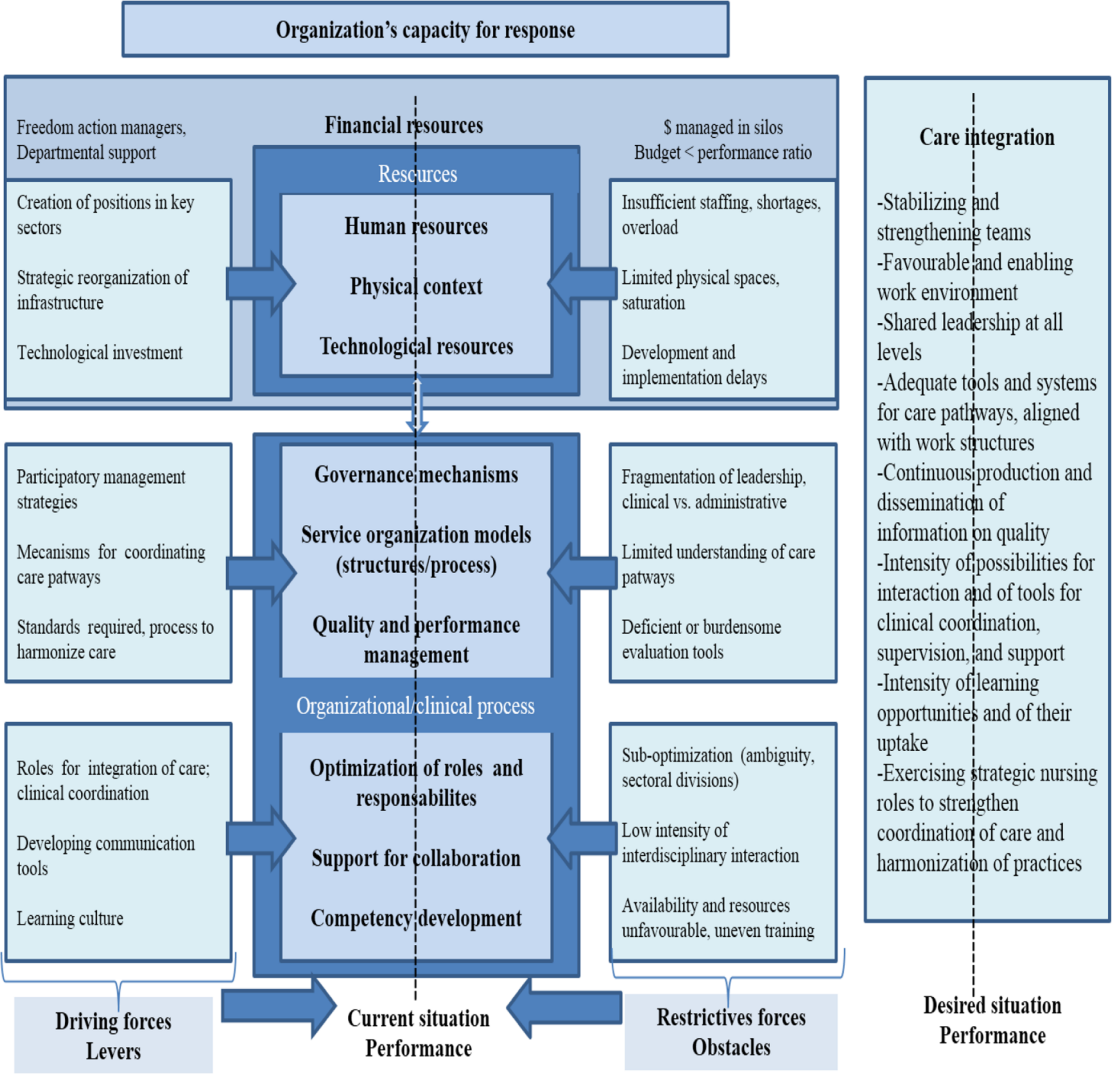
Strengths of and impediments to an organization’s capacity for response

Participants referred to two categories of factors that influenced the organization’s capacity to implement and sustain a nursing practice that met the requirements of integration. The first had to do with availability of organizational resources, and the second involved deployment of a set of organizational and clinical-administrative processes (Fig. 4).

### Organizational resources

With regard to resources, three key factors were seen as having a determining influence on nursing practices to support integrated care, either serving as catalysts or generating a variety of constraints.

#### Human resources

Participants pointed out that certain investments in human resources played a key role in the organization’s capacity to put in place various structures to foster integration of care. Examples of new structures that required appropriate human resources staffing included the single-window access point (a single gateway for patients in the healthcare system, where they are assessed and guided according to their needs) for mental health services, the breast health clinic (POS), and the smoking cessation clinic (COPD). These new investments also resulted in the deployment of specific professional roles (e.g. nurse navigators in palliative care, care for the elderly, and oncology; mental health liaison nurse, etc.), the opening of new nurse positions in targeted areas (need for better integration of patient care), and support for new continuing education activities for personnel.

Despite these investments, participants emphasized two factors that, under current conditions, impeded a nursing practice oriented toward care integration. The first was that, in their view, there were not enough nurses to meet the demand. According to participants, having enough nurses is essential to respond effectively to patients’ complex needs and ensure the stability required for continuity of care. When personnel is insufficient, as was reported in several programs, nurses are over-extended, both in terms of quantity and complexity of care, and do not have enough time to provide the full range of services to each patient. In the medical unit, nurses reported they sometimes had to limit themselves to looking after patients’ basic care needs due to lack of time, and that they would neglect relational activities, for instance, to concentrate on complex or prescribed care. Several participants noted that staff shortages also compromised access to care at various points along the care continuum.

*“A workload reduction would help promote a practice of more continuous care for patients. We could be on top of everything, it would be easier”* (MPOC) *(free translation)*

*“Sometimes I’m all over the place, I have to refocus on my objectives, and my boss helps a lot with that… The staff is starting to get tired and worn out. Two years ago, 25 patients a day in chemo was a big day, now it’s around 47-48 patients…”* (POS) *(free translation)*.

In the MHS pathway, this shortage of personnel was associated with long wait lists for primary care services. Staff shortages were also associated with nurses’ lack of availability to attend intra- or interdisciplinary team meetings, continuing education activities, or discussions with colleagues—all elements that foster integrative and collaborative practice.

A second obstacle had to do with team instability, manifested most notably by frequent changes in personnel, numerous replacements, and intensive use of external labour. These instability factors were associated with nurses’ difficulties in becoming fully integrated into the team, taking part in unit activities, establishing relationships of trust with other members of the intra- or interprofessional teams, and mastering the operational aspects of care—all elements that can compromise care continuity. Replacement staff do not necessarily know the different resources that are available. Thus, an agency nurse might not be as quick to refer a hospitalized patient who wants to quit smoking to the smoking cessation clinic, or to call upon the palliative care nurse navigator to coordinate a patient’s transfer to the palliative care centre. Replacement personnel would also have more difficulty in using the nursing treatment plan, a key tool for monitoring and coordinating care from one shift to another, one care location to another, or even one professional to another.

#### The physical setting for care provision

The creation of local health networks specific to the Quebec context has significantly modified the spatial configuration of services, with impacts for both patients and professionals. According to nurse participants, the modernization and expansion of the hospital and renovations to various care settings had made it possible to create the spaces needed for various new strategic services in which nurses are fully involved and which should foster greater integration. Setting up the infrastructure needed to implement a centralized wait list, for example, helped create a single point of entry for health services for certain target patients (e.g. MHS, POS, ASE). Developing this infrastructure (offices, telephone lines, information systems, documentation) placed nurses at the centre of the care integration process by giving them a set of resources with which to manage requests for services and patients’ records, and to conduct assessments, set priorities, initiate treatments, and direct patients toward other appropriate services. The implementation of a system to manage COPD patients was another example of how providing certain material conditions, such as organizing physical spaces to accommodate all care team members (nurse, respirologist, respiratory therapist) and supplying all the necessary material and equipment, had facilitated various nursing interventions. These included systematically applying pulmonological assessment protocols, using digital tools to monitor patients, and developing educational capsules for patients. For participants, the various arrangements involving new construction or renovation and investments in certain equipment (e.g. meeting rooms, computer equipment, consultation rooms, and rooms for examinations or specific interventions) were interpreted as an appropriate response to the different needs associated with functioning as a network: physical spaces for meetings and conversations, communication and monitoring from a distance, and intra- and inter-establishment interactions.

Despite these investments, various factors related to the physical environment were perceived as important constraints to the integration of services provided by nurses. The limitations of physical spaces, which in many cases were considered too small and poorly adapted, made it difficult not only to accommodate patients, but also to deploy personnel optimally and to gather all members of the interdisciplinary team around patients. Lack of space in the oncology centre, for example, limited the number of patients that could be seen daily, the quantity and type of services that could be provided, and even the number of nurse navigators and other professionals who could practice on the unit at the same time. Nurses had to go from one place to another in the organization to ensure continuity of care to patients, or to attend team meetings or training activities. Furthermore, the merger of several organizations that were geographically dispersed inevitably resulted in physical distance between professionals in the different missions (e.g. primary care, hospital care, residential care for the elderly) who were expected to work together. According to the participants, this geographical dispersion of facilities, with the associated dispersion of equipment, patients, and personnel, posed a significant challenge for communication, complicated interdisciplinary activities, forced both patients and staff to navigate among the different facilities, and ultimately made continuity of care more difficult to achieve.

*“We don’t have enough space in the oncology unit, because the amount of traffic has increased significantly, the clinic is overloaded. Even if we wanted another nurse navigator, there wouldn’t be any room for her. The care is less personalized, there is more assembly-line work, and the wait times are too long (6-7 hrs for 30 min. of treatment). Luckily, there is an expansion project underway. All the services will be located together and there will be less patient movement. Which is much appreciated by the patients and professionals”* (POS) *(free translation).*

#### Technological resources

In terms of technological resources, participants pointed out several assets available to the organization for fostering nursing practice to support integrated care, reinforce communication and exchange with other professionals, and mitigate the negative impacts of geographic dispersion. The intranet, widely deployed and accessible across the organization, was associated with an integrative function because of the opportunities it offered, both clinically and administratively, as well as for training. It was presented as a lever for ensuring liaison among different members of the care team, communicating information in a timely manner, and promoting a high quality of practice. For example, for the nurses working in ASE, whose patients have numerous and complex health problems, the intranet was a useful consultation tool for exchanges on clinical practices and for promoting best practices. A second asset mentioned was the progressive implementation of computerized medical records. Because it created conditions that facilitated data organization, access, and circulation, the computerized medical record was widely considered to be a lever for encouraging care coordination among nurses and facilitating interactions with other members of the interdisciplinary team. For example, a designated nurse used computerized medical records to identify patients who were high users of emergency services, then made the necessary arrangements to coordinate and mobilize the interdisciplinary team to respond optimally to these patients’ needs for care and services. A third asset consisted of computer-based resources, such as specialized software, that provided nurses with monitoring and assessment tools to ensure better care coordination. A software program developed especially for COPD patients and used by nurses in FMGs and ambulatory care centres helped systematize patient assessment and monitoring and provided a shared tool for intervention and coordination used by nurses, respiratory therapists, and physicians.

The possibilities associated with these technological resources were, however, limited by certain obstacles. Nurses complained about slow deployment of computerized medical records, about obsolescence of the technological tools, which were not updated quickly enough to keep up with technological advances, and about inadequate support for professionals who need to use these technologies (training, availability of support technicians, time required to use them, etc.).

*“Computerized records have not enabled technological support for a daily watchlist available to practitioners in the network. We are dependent on maintaining the ‘pop-up’ of information, there is no mechanism to alert the team when someone arrives in emergency”* (MPOC) *(free translation)*.

In all, participants strongly associated the organization’s level of investment in the three types of resources (physical, technological, and human) with the creation of conditions fostering greater integration of the care provided by nurses. Such investments depended very much on the budget allocated to the organization. The MSSS or the Health and Social Services Agency (ASSS) provided funding for specific projects (e.g. development and implementation of a clinical project). However, participants’ general perception was that the organization had very little room to manoeuver when it came to investing in resources required for the integration project. They saw this underfunding as a major cause of the various structural obstacles to integration mentioned above: physical space constraints, insufficient personnel for key care-provision functions, obsolete technologies.

### Clinical-administrative processes

Six main process-related factors were seen as having a determining influence on nursing practices to support integrated care, involving both administrative (governance mechanisms, service organization, quality and performance management) and clinical aspects (introduction of care integration roles, support for interdisciplinary team work, capacity strengthening).

#### Governance mechanisms

With respect to governance, participants first highlighted efforts made by the organization to promote a shared vision of care integration and to set some broad orientations. In particular, these efforts took the form of activities to involve different groups, including nurses, in developing an organizational project and a strategic plan for the institution. Such activities included, for example, days of reflection, consultation meetings, training sessions, collective problem-analysis approaches, and problem-solving discussion groups or workshops. Another strategy seen as facilitating was the implementation of governance structures to ensure collective responsibility for the integration project and allow shared leadership to be developed at all levels of the organization. This resulted in participative management approaches that included, among other things, interdisciplinary workshops, working committees at different organizational levels involving employees from different shifts and points of service, the use of various spokespersons representing different groups in the organization, and a relative increase in clinical-administrative meetings through which the contributions of the different organizational groups could be solicited. Nurses in the different pathways were engaged to varying degrees in the process of carrying out the clinical project. The Nursing Directorate itself (which includes the Director of Nursing at the HSSC and plays a key role in all strategic decisions related to the organization and delivery of care and services) invested in promoting a cross-disciplinary view of patient care, using clinical nursing consultants. At a more operational level, nurses were directly involved in developing the care pathways. The mental health liaison nurse developed a process for emergency room triage assessment of patients with mental health problems that enabled them to be referred more rapidly to the appropriate service. A clinical nurse consultant with a master’s degree developed and implemented a project to evaluate high users of emergency room services, with mechanisms for interdisciplinary follow-up of these patients (COPD). Participants interpreted these various activities as developing an integration-oriented culture that was based on providing more opportunities for interactions among the actors (health professionals and managers), creating a variety of spaces for collaboration and learning, and involving the actors in various collective learning processes.

Nevertheless, despite the efforts that had been made, participants noted various gaps in governance that explained the delays seen in service integration. In contrast to the ideal of shared leadership originally espoused, leadership was fragmented among different departments whose interventions were not sufficiently coordinated, which impeded care continuity in the care pathways. One respondent in mental health explained, for example, that for a mental health patient with multiple pathologies, the departments of physical and mental health needed to work together, which was not always the case. Moreover, while the subcultures of certain units or institutions (e.g. CLSCs, primary care mental health services) are very much accustomed to interdisciplinary work and referral mechanisms, other settings, such as hospitals, have maintained a culture of working in silos. Another problem mentioned by several participants was the different actors’ varying levels of involvement in the change process, depending on their settings. Some nurses in the COPD pathway working in the medical unit expressed frustration at feeling marginalized in these change processes.

*“It’s a big machine, things are complicated. Before, it was smaller. Was it better? I don’t know. Sometimes the trouble arose from questions of values between groups. Adjustments have to be made on all sides; departments, managers, coordinators, practitioners. There are many small cultures that have to be plugged in* (ASE) *…Things change, there are links that form, we weren’t as closely tied to the CLSC before. In some ways, it’s also gotten more weighed down. People don’t quite know who to refer to anymore when there’s a need”* (MHS) *(free translation)*

They reported that they had not been involved in any way in the change process, not due to lack of interest, but rather because they had not been given the opportunity. Other participants noted that some professionals were reluctant to engage in the change processes for various reasons: misgivings, negative perceptions of change, lack of information. Rumours circulating to the effect that a number of nursing positions in secondary care (e.g. intermediary resources) in the MHS pathway would be cut in order to open nursing positions in primary care (e.g. home care) naturally raised concerns among the nurses involved.

#### Service organization models

In this area, participants referred to various service reorganization processes and clinical-administrative instruments that were associated with better patient management and with the capacity to provide a better-coordinated range of services. They spoke about three types of processes. The first were processes to strengthen links between primary and secondary care teams. One example was a program of shared services between the two levels. There was a significant service to support general practitioners in the territory, wherein a contact psychiatrist helped clarify diagnoses and made recommendations regarding medical treatments. The second type involved processes and tools to facilitate patient navigation of the system and refer them to the most appropriate services. This involved developing, for example: detailed maps of MHS pathways; protocols to guide follow-ups and transfers to appropriate services; service provision guidelines for the oncology nurse navigator or for nurses managing the wait list; mechanisms to clarify the roles of professionals providing ASE services; and intervention instruments, such as decision-support tools and intervention plans tailored to the patient’s condition for ASE nurses. The third type of process consisted of grouping together in one location a set of complementary services provided by an interdisciplinary team to respond more effectively to the needs of certain types of clienteles (e.g. intake clinic in respirology, palliative care centre, general practitioner/psychiatrist shared services program). However, certain obstacles were mentioned as factors that impeded the above-mentioned processes.

*“There again, everything goes to secondary. There’s an issue there. So, do I try to make secondary care more competent to provide services to patients with serious conditions, to make it functional and efficient? If so, that’s excellent, but what do I do with primary care that then isn’t keeping up? This takes some thought. It’s the major challenge”* (MPOC) *(free translation)*

The obstacles included, among others: resources being concentrated in secondary care, slowing the deployment of primary care services; a lack of knowledge about the range of existing services, both among patients and among the professionals themselves; professionals’ scope of practice often being defined more by their position or assignment to a given unit than by patients’ needs; and a narrow conception of care pathways that only partially covers patients’ real needs. The inclusion criteria determining patients’ access to certain care pathways were seen as limiting the management of a variety of situations, especially for patients with multiple pathologies. For instance, professionals working in the COPD pathway could not manage on their own the substance use problems presented by some of their patients.

#### Quality and performance management

In this area, participants listed three facilitating factors as levers used to foster nursing practice aligned with organizational orientations regarding care integration. The first had to do with the professionals’ efforts to align care with best practices. For example, in ASE the nurses and physiotherapists adopted the same practices for mobilizing residents, based on evidence drawn from the literature. In another case, a pressure sore protocol for patients receiving terminal care at home was adopted by the key professionals involved: nurses and occupational therapists. The second factor had to do with the organization’s vigilance in compiling statistics on several nursing-sensitive quality indicators. These indicators included, among others: smoking cessation rates (nicotine addiction clinic); numbers of hospitalizations and lengths of stay; effectiveness of patient education activities in relation to symptom management (COPD clinic); levels of service use (high users of emergency services); numbers of consultations (oncology nurse navigator, primary care mental health nurse, ASE wait list managers); and occupancy rates for stretchers in psychiatric emergency or in palliative care. The third factor involved reports that were required to be submitted to various internal and external bodies (regional agency; accreditation bodies; professional associations; local complaints commissioner; users’ committee; quality assurance committee; council of physicians, dentists, and pharmacists; council of nurses; etc.) regarding services, including those provided by nurses. Participants associated several of these reporting instruments with a desire to assess nurses’ performance, whether directly or indirectly: staff and patient surveys as part of the accreditation process; patient satisfaction questionnaires; stretcher occupancy rates in MHS; and incident/accident reports.

According to participants, however, implementation of these performance management tools masked several significant limitations. The instruments (measurement tools, information systems) used to measure the quality of nursing services were still considered inadequate. Several new roles had been introduced (e.g. palliative care nurse navigator, COPD liaison nurse) and had not yet been formally evaluated.

*“Unfortunately, we know that performance associated with stretcher occupancy rates is more important than the level of performance in quality of care. A drop in performance could mean we risk losing stretcher to [physical] medicine for example. In all that, is the quality of patient care really being evaluated?”* (MHS) *(free translation)*

The gap between needs and resources, as well as the pressure associated with certain performance requirements (mainly in terms of service volumes), generated significant stress for the personnel. In the MHS ambulatory clinic, a key performance indicator was the number of patient assessments performed daily. The oncology unit’s recognition as a regional cancer centre depended, among other requirements, on the number of patients managed by the nurse navigator.

#### Introduction of care integration roles

In this area, participants referred repeatedly to several new nursing roles that were considered integrative roles: liaison nurse, nurse navigator, nurse clinician consultant, case manager, network professional. These roles were seen as levers for improving the coordination of services for certain target patient groups, for supporting them in transitioning among different levels of care, for ensuring more rapid access to certain professional resources, and for optimizing the use of those services through more accurate referrals of patients needing them. The oncology nurse navigator contributed to care integration by orchestrating the oncology patients’ medical records. At work, she was available to respond to her patients’ needs, made connections between the various health professionals (e.g. multidisciplinary team) involved in patients’ care, and ensured that patients were appropriately followed both outside and inside the department of oncology, as needed. The liaison nurse in the medical unit contributed to the COPD care integration process by mobilizing necessary resources and linking with professionals to ensure patients continued to be followed, mainly after discharge or upon returning home. The ASE nurse clinician consultant looked after the quality of care provided to elderly patients by all institutions in the network. Based on her comprehensive overview, she ensured that nursing services to these patients were optimized; she did clinical coaching and developed tools, protocols, and collective prescriptions; and she trained teams in collaboration with physicians and pharmacists.

However, participants noted many obstacles to full deployment of these new roles. Several participants reported that a significant proportion of clinicians and managers had a poor understanding of these roles and their potential, resulting in under-utilization of the persons in these roles. Another major difficulty had to do with financial constraints, which often made it difficult to ensure the sustainability of positions associated with these roles.

*“I could see myself intervening more in complex care situations. I am not being used at full capacity. Since I can move between facilities, I could easily act as a bridge between situations, which is not often enough the case”* (POS) *(free translation)*.

Finally, because these roles were not very standardized in terms of how they were defined and enacted, they varied considerably depending on the specifics of different settings and professionals. The resulting ambiguity added to the complexity of collaborating with other members of the interdisciplinary team, thus impeding integration of care.

#### Support for collaboration

On this theme, participants referred to four types of activities they perceived as being levers for promoting collaboration among team members and for coordinating their interventions more effectively.

The first type of activity involved strengthening methods of communication and transmission of clinical information. The progressive deployment of computerized medical records in all the CSSS settings was perceived as an important resource for transmitting clinical information. In addition to the computerization of medical records, various activities were used to develop new tools for exchanging information, making referrals, and following patients. The second type of activity consisted of putting in place shared working tools to improve patient management: progressive implementation of the nursing treatment plan, in which all nursing team interventions were recorded; development of standardized protocols and care tools based on best practices, involving collaboration between physicians and nurses. The third type consisted of increasing opportunities for intra- and interprofessional interaction by making more dedicated spaces available; holding monthly meetings and admission/discharge meetings in the ASE pathway; attendance at regional interdisciplinary round tables by nurses in the MHS pathway; and initiatives that were currently being introduced to hold more systematic interdisciplinary and team meetings in the MHS pathway. The fourth type of activity consisted in creating or strengthening roles dedicated to clinical coordination. Nurse clinician consultants, nurse navigators, and liaison nurses were working to support teams in adopting new practices and working tools and in coordinating their interventions more effectively. The RISPA nurse (*Réseau intégré de soins pour la personne âgée*—Integrated network of care for the elderly) was mandated to coordinate the activities of all professionals involved in providing care to elderly patients. An oncology nurse was assigned to manage all transfers to palliative care and to ensure coordination among the various professionals and managers involved in these transfers.

According to participants, the possibilities associated with these various activities were constrained by several factors: a lack of human resources; difficulties encountered by the people in these positions making themselves available for team activities, such as interdisciplinary meetings; a lack of preparation for interdisciplinary work; difficulties encountered by some professionals in interacting or sharing information with others; variability in the opportunities available to different professionals depending on their work context (e.g. fewer opportunities for interaction among persons working evening and night shifts, or working in relative isolation—such as the smoking cessation nurse—or even those in care units where the culture of holding interdisciplinary meetings was less developed).

*“One mentality and philosophy that should be changed would be to remove the words ‘my patient’ from our vocabulary. It’s ‘our patient’ or ‘the patient you referred to me’… Also, to see other professionals as complementary, rather than as threatening. There are people who want to do everything with their patient, rather than using the strengths of everyone, which would enable the person to move forward … and bring other ideas and intervention possibilities to our team discussions”* (MHS) *(free translation)*.

#### Capacity strengthening

In this area, participants referred to the organization’s investments in two main types of activities that supported nursing practices to support integrated care. First, the implementation of care pathways gave nurses access to several new learning opportunities. Interdisciplinary meetings, involvement in the development of new tools and service models, and participation in working committees were perceived as opportunities to acquire knowledge and develop competencies needed to implement more integrated care. Along the same lines, participants highlighted the support received in communities of practice and in intra- and interdisciplinary co-development groups. In POS, for example, the tumour board, made up of members of the medical, professional, and nursing team, provided regular opportunities to discuss complex cases and reach consensus on the best treatments. Second, the organization had engaged nurse clinicians, nurse consultants, nurse navigators, and liaison nurses in a series of activities aimed at offering formal training activities, promoting self-study activities, and providing clinical support to nurses, such as through coaching or mentoring. Nurses with a particular expertise were invited to present training capsules or lunch conferences on topics of interest related to best practice development.

*“Certainly, having moments to think, to review your situation, and to create the opportunity to share with other practitioners, it’s amazing how enriching it is, but it’s not easy within the organization or for a nurse in the SAD [home support] who has to answer for her day’s caseload”* (MHS) *(free translation)*.

The main obstacle to implementing these activities was nurses’ lack of availability, in contexts often characterized by inadequate staffing or excessive workloads. In addition, because of the physical distances involved, training tended to be designed for each setting rather than around care pathways, making standardization more complex.

## Discussion

A series of driving forces (demographic, epidemiological, social, and economic) is pushing a transformation of healthcare system practices toward greater integration of care. Organizations’ capacity for response in terms nursing practices to support integrated care is a black box that until now has hardly been explored. This study has opened that black box and shown that this capacity for response can be expressed through two types of complementary interventions that involve: 1) investing in a set of key resources and renewing organizational structures; and 2) deploying a series of organizational and clinical-administrative processes. This analysis was conducted based on the study of an HSSC confronted with the same challenges faced by most HSSCs in Quebec. In addition, all health institutions at the national and international levels confronting fragmentation of care and the need to rationalize care and services through better integration are faced with these challenges. The case was analyzed with regard to the investments made by the organization, the strategies adopted, stakeholders’ perceptions of these strategies, and gaps still to be addressed. The results, summarized in Fig. 4, show that the organization’s capacity for response to the driving forces calling for more nursing practices to support integrated care is mitigated.

In terms of resources, integration efforts took the form of investments in implementing and staffing new strategic services, re-organizing physical infrastructures, and deploying new technological resources. According to study participants, however, these investments had limited impacts in several study environments and were not sufficient to offset a persistent set of constraints related, among other things, to insufficient human resources, slow deployment of computerized medical records, lack of space to accommodate interdisciplinary teams, and the geographic dispersion of facilities. Moreover, the current context of the healthcare network is marked by massive budget cuts. These conclusions highlight the importance of investing in the work environment as one of the fundamental levers that must be fully activated to create a supportive context for integrative practices. Work environment refers to a set of properties having to do with the arrangement of infrastructures, the physical and spatial organization of work, the availability and organization of material and technological resources, human resources staffing, and the availability of a set of psychosocial resources [22, 23]. Various writings on the topic of structural *empowerment* discuss the impact of these structural factors on nurses’ provision of services [24, 25]. These factors pertain to nurses’ access to a variety of conditions and resources that support them in carrying out their tasks: information, sources of support in the organization, material resources, and possibilities for professional advancement. Two decades of research on Magnet hospitals have shown that a work environment in which there is adequate staffing and access to a variety of sources of professional support is associated with improved services to patients [26]. Other studies on high-performing systems have shown that they are characterized by access to a certain number of resources (technological, informational, human) that give them the ability to innovate in their service production processes [27]. All of this converging evidence shows that the lever provided by the work environment was not fully activated in the case examined in the present study, given the persistent problems.

In terms of organizational processes, the integration efforts made in the study case resulted in a variety of activities aimed at renewing governance, introducing participative management approaches, improving the flow of care pathways, and promoting continuous quality improvement. Here again, these efforts were offset by difficulties related to fragmented leadership, poor knowledge about the care pathways on the part of both patients and care providers, and shortcomings in quality monitoring.

These conclusions highlight three levers that were only partially activated in this service transformation process: the exercise of shared leadership; the systematization and instrumentation of care pathways and their alignment with the work structure; and the production of information on quality. With regard to leadership, a study that examined, among other things, the topic of clinical governance has shown that high-performing health organizations are characterized not only by a strong management team, but also by shared and collective effort, and a joint commitment by clinicians and managers at all levels to steering and implementing improvement initiatives [28]. Regarding systematization of care pathways, using such a lever gives organizations the possibility of standardizing practices, programming and structuring clinical interventions more effectively, and aligning them with the best evidence and practices to optimize patient pathways [29]. This involves being able to develop and implement standardized protocols on a large scale, to develop and implement shared intervention tools, and to adopt and institutionalize proven guidelines and practices, supported by strong evidence. With regard to producing information on quality, such a lever makes it possible to generate information needed to substantiate the analysis of care processes, diagnose problems early, and implement evidence-based solutions [30], thereby laying the foundations for learning systems in which clinicians and managers have access to the information needed to assess the quality of their services and to ensure continuous improvement [30]. This case analysis showed a definite will to activate each of these three levers, but with interventions that did not have the full impact needed to overcome obstacles and achieve the desired results.

In terms of clinical processes, the integration efforts led to a variety of activities aimed at developing new roles, promoting collaboration among clinicians, and strengthening the capacities of service providers. However, advances in this area were offset by delays in the deployment of new roles, a continued low intensity of interdisciplinary interactions, and practice conditions that did not leave professionals enough time to engage in learning activities and capacity strengthening. These findings indicate that two levers were not used to their full potential in the case examined: reinforcement of coordination, supervision, and clinical support mechanisms; and capacity strengthening.

The lever of clinical coordination reflects the notion of clinical leadership and refers to implementing an infrastructure that would support teams in adopting new work practices and would also promote intra- and interprofessional collaboration [28]. It involves consolidating the leadership of various intermediate managers (program heads, coordinator) and mobilizing professionals in their role of facilitating, coordinating, and supporting their peers (multiplying agents, expert clinicians, patient navigator) [30]. Such a coordination infrastructure can serve as a platform to create more frequent opportunities for interaction and exchange among team members (interdisciplinary meetings, case discussions, team meetings), ensure the communication and transmission of clinical information, implement tools to reinforce interdisciplinary work (e.g. interdisciplinary assessment forms, interdisciplinary intervention plans). This lever also refers to integrator roles that go by a variety of names (e.g. liaison officers, case managers, patient navigators), providing many opportunities to reinforce the coordination of services for target user groups, support them in their transitions among different care levels, and ensure more rapid access to certain professional resources [31].

The capacity-strengthening lever refers to strategies and teaching methods that should be deployed to support transformation processes. These investments in personnel, their professional development, and learning processes can help improve not only care provision and patient or client satisfaction, but also the satisfaction of professionals [32]. The amounts invested can also be recovered thanks to increased efficiencies generated by improvements in management processes and service provision.

The results of this study should be interpreted in light of two main limitations. The first is that this analysis was based on a single case study. Despite the richness of this case and the similarity between the issues seen here and those facing the great majority of HSSCSs in Quebec, we cannot exclude the possibility that other levers might have been identified if the sample had been expanded to include a wider variety of institutions and settings.

The second limitation concerns the essentially perceptual nature of the data upon which the study was based. While this made it possible to address the questions through multiple stakeholders’ viewpoints, some of the factual information provided by participants was not verified. Despite these limitations, this study shed light on the main levers that organizations can employ to encourage the implementation and institutionalization of integrative nursing practices in their efforts to lessen the current fragmentation of services.

## Conclusions

This study indicates that progress in this area will require a combination of strategies that involve mobilizing several complementary levers: a supportive work environment; shared leadership; systematization and instrumentation of care pathways, and their alignment with the work structure; production of information on quality; reinforcement of coordination, supervision, and clinical support mechanisms; and capacity strengthening. This study highlights the fact that progress in implementing these more integrative practices will necessarily require rethinking the deployment of certain organizational resources and structures. However, beyond structural aspects, fundamental changes are also needed across all clinical and organizational processes.
